# Oral Cephalosporin-Induced Anaphylaxis Presenting With Severe Tachycardia: A Case of Microdosed Epinephrine Over Standard Protocols

**DOI:** 10.7759/cureus.88683

**Published:** 2025-07-24

**Authors:** Anupama R S, Rohan Krishna NK, Sathya Lakshmi Mekkoth, N Sridhara, Shivaraj K

**Affiliations:** 1 Emergency Medicine, Fortis International Hospital Rajajinagar, Bengaluru, IND; 2 General Practice, Fortis International Hospital Rajajinagar, Bengaluru, IND; 3 Cardiology, Fortis International Hospital Rajajinagar, Bengaluru, IND; 4 Internal Medicine, Fortis International Hospital Rajajinagar, Bengaluru, IND

**Keywords:** atypical anaphylaxis, cephalosporin allergy, drug-induced anaphylaxis, facial angioedema, hypersensitive reaction, ige-mediated hypersensitivity, intravenous epinephrine, micro-dosing adrenaline, severe tachycardia with normotension

## Abstract

Anaphylaxis is a severe, rapidly progressing hypersensitivity reaction that requires prompt recognition and administration of intramuscular epinephrine. While guidelines recommend fixed-dose intramuscular epinephrine regardless of heart rate or blood pressure, there are situations where this approach may carry risks. We present the case of a 27-year-old patient with no prior comorbidities, including asthma, allergies, or cardiovascular conditions, who developed sudden breathlessness, generalized urticaria, and swelling of the face and lips shortly after taking oral cefpodoxime, a third-generation cephalosporin, prescribed for a febrile illness at a local hospital. On arrival at our hospital, the patient was conscious and alert, with an oxygen saturation of 87% on room air, a heart rate of 169 beats per minute, and a blood pressure of 100/60 mmHg. The ABCDE (Airway, Breathing, Circulation, Disability, Exposure) protocol was followed, and supportive care including oxygen, nebulized bronchodilators, and intravenous fluids was provided. Given the clear signs of anaphylaxis with marked tachycardia but no hypotension, there was a dilemma regarding the administration of standard intramuscular epinephrine due to concerns about worsening tachyarrhythmias. Under close monitoring, carefully titrated microdosed intravenous epinephrine was administered using a dilution protocol, resulting in rapid symptom improvement without cardiovascular instability. The patient was admitted to the intensive care unit for monitoring and supportive care and was later discharged in stable condition with oral antihistamines, corticosteroids, and clear instructions on allergen avoidance to prevent future emergencies. This case illustrates the need for clinical judgment when strict adherence to guidelines may pose a risk and demonstrates the role of individualized microdosed intravenous epinephrine in managing anaphylaxis in patients with significant tachycardia and normotension.

## Introduction

Anaphylaxis, first described by Charles Richet and Paul Portier in 1902, is an acute, serious, systemic, and life-threatening IgE-mediated hypersensitivity reaction [1]. It typically manifests with a combination of urticaria, pruritus, angioedema involving the lips, tongue, or throat, respiratory distress in the form of wheezing, stridor, dyspnea, hypotension, syncope, abdominal pain, vomiting, and incontinence [2,3]. These symptoms are the result of a cascade of inflammatory mediators and cytokines released from mast cells and basophils following exposure to an allergen.

Common elicitors include foods, insect venom, and medications, with drugs accounting for a significant proportion. In a retrospective study from Odense University Hospital involving 226 patients, medications were implicated in 41.1% of anaphylactic episodes [4]. While the incidence of anaphylaxis globally is estimated at 50-112 cases per 100,000 person-years, the true prevalence is likely underestimated due to underrecognition and misdiagnosis in clinical settings [5].

Although anaphylaxis is primarily a clinical diagnosis, certain laboratory investigations can provide supportive evidence. Serum tryptase levels, when measured within 2-3 hours of symptom onset, may help confirm mast cell degranulation and strengthen the diagnosis in ambiguous presentations. Allergen-specific IgE assays and skin prick testing are more useful post-reaction, aiding long-term management and avoidance strategies [6,7].

The cornerstone of anaphylaxis treatment is the timely administration of epinephrine, which activates α1, β1, and β2 adrenergic receptors. This results in vasoconstriction, increased cardiac output, and bronchodilation. Current guidelines recommend a dose of 0.3-0.5 mg administered intramuscularly into the anterolateral thigh, with repeated dosing every 5-15 minutes as needed [8].

While intramuscular epinephrine remains the cornerstone of anaphylaxis treatment, its use should be guided by the patient's clinical context. Most protocols recommend a fixed-dose approach regardless of heart rate or blood pressure. However, in certain situations where the patient presents with severe tachycardia and normal blood pressure, this approach may need to be reconsidered. In these cases, the risk of inducing arrhythmias or placing additional strain on the heart becomes a valid concern. In our patient, who arrived with marked tachycardia and normotension, standard intramuscular epinephrine was considered high risk. Instead, an individualized approach was adopted using careful titration and microdosing of epinephrine. This allowed for effective symptom relief while reducing the potential for cardiovascular complications, highlighting the importance of flexibility in clinical decision-making when managing anaphylaxis.

## Case presentation

A 27-year-old female with no prior comorbidities presented to the emergency department with complaints of breathlessness, generalized urticaria, itching, and swelling of the face and lips. Earlier that morning, she had presented to a local clinic with a two-day history of fever accompanied by myalgia and generalized weakness, which had persisted despite taking paracetamol. She was prescribed cefpodoxime 200 mg, a third-generation cephalosporin, along with paracetamol and supportive medications. Shortly after taking the antibiotic, she developed itching, urticaria, and facial and lip swelling, prompting her visit to the emergency department. She reported no chest pain, cough, palpitations, syncope, calf pain or swelling, recent travel, prior episodes of similar reactions, known drug allergies, asthma, vomiting, diarrhea, or urinary complaints.

On presentation, the patient was conscious and alert. The ABCDE (Airway, Breathing, Circulation, Disability, Exposure) protocol was then followed. The airway was assessed and found to be patent. Breathing was compromised, with a respiratory rate of 40 breaths per minute, oxygen saturation of 87% on room air, and bilateral rhonchi audible on auscultation. Oxygen therapy was initiated at 2 liters per minute via face mask, followed by nebulized bronchodilators to address respiratory distress. On circulatory assessment, the patient had a heart rate of 169 beats per minute and a blood pressure of 100/60 mmHg. Intravenous access was established, and baseline blood investigations were sent. An infusion of normal saline was initiated at 100 mL per hour. Disability assessment confirmed that the patient was alert and oriented. On exposure, a generalized urticarial rash was noted, and axillary temperature was recorded at 103°F. Although the initial presentation included fever suggestive of an underlying viral illness, the elevated temperature at admission was interpreted as part of the systemic inflammatory response to the acute anaphylactic reaction following antibiotic ingestion.

Given the sudden onset and close temporal association with the drug, anaphylaxis was considered the primary diagnosis. However, due to the presence of fever and tachycardia, differential diagnoses such as sepsis, dengue, thyrotoxicosis, pulmonary embolism, and asthma exacerbation were systematically evaluated and excluded through clinical assessment and relevant investigations (Table 1).

**Table 1:** Differential Diagnoses. CXR: chest X-ray; TSH: thyroid-stimulating hormone

Differential	Reason Considered	Exclusion Findings
Anaphylaxis	Temporal relation to drug, urticaria, angioedema	Confirmed
Sepsis	Fever, tachycardia	Normotension, clear CXR, negative cultures
Dengue	Fever, tachycardia	NS1/IgM negative
Thyrotoxicosis	Tachycardia, fever	Normal TSH
Pulmonary embolism	Tachycardia, hypoxia	No pleuritic pain, clear CXR
Asthma exacerbation	Wheeze, dyspnea	No prior history, urticaria present

**Figure 1:**
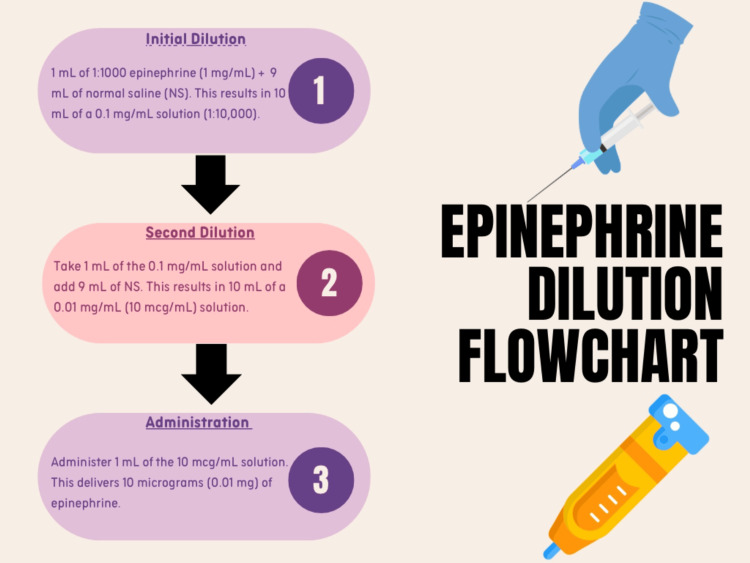
Stepwise Epinephrine Dilution and Administration Protocol for Microdosed IV Use in Anaphylaxis NS: normal saline

Given the significant tachycardia, instead of administering the standard intramuscular epinephrine, cautious microdosed intravenous epinephrine was initiated under continuous monitoring to mitigate the risk of inducing or worsening tachyarrhythmias. Similar individualized strategies have been advocated in emergency care literature, particularly in settings where the hemodynamic profile makes standard intramuscular dosing potentially hazardous [9]. The detailed dilution protocol used for administering the microdose epinephrine is outlined in Figure 1.

Following administration of microdosed intravenous epinephrine, the patient showed clinical improvement, with relief in respiratory distress and normalization of oxygen saturation. The baseline and post-stabilization vitals have been summarized in Table 2.

**Table 2:** Baseline and Post-Stabilization Vitals.

Parameter	At Presentation	Post-Stabilization
Heart rate	169 bpm	100 bpm
Blood pressure	100/60 mmHg	115/75 mmHg
Respiratory rate	40	18
SpO₂ on room air	87%	98%

In view of the initial respiratory compromise requiring oxygen support, epinephrine administration, persistent tachycardia, and the risk of a biphasic anaphylactic reaction, the patient was admitted to the intensive care unit for close monitoring and supportive care.

**Figure 2:**
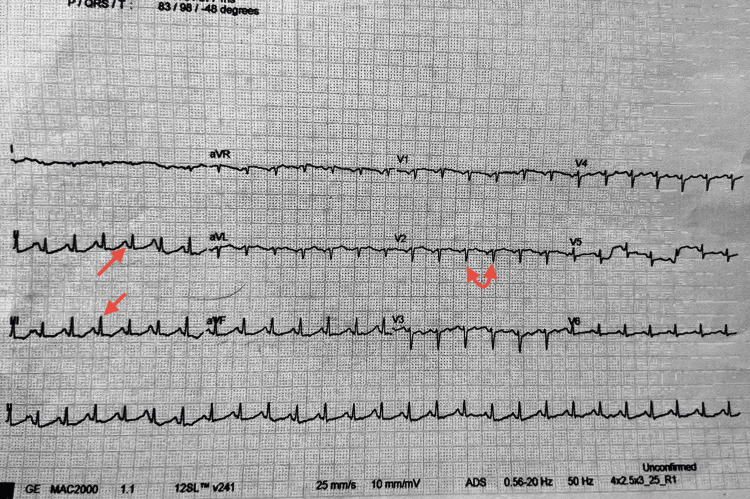
A 12-lead ECG showing sinus tachycardia with visible P waves, narrow QRS complexes, and a regular rhythm at presentation (red arrows).

A 12-lead electrocardiogram performed on admission (Figure 2) showed a narrow complex tachycardia with a ventricular rate of approximately 160-170 beats per minute. Clear P waves were visible before each QRS complex, confirming a sinus origin. The QRS complexes were narrow, with normal axis and morphology, and the rhythm was regular. There was no evidence of ST-segment elevation or depression, T wave inversion, or other ischemic changes. These findings were consistent with sinus tachycardia, likely secondary to the acute anaphylactic reaction and systemic stress response.

**Figure 3:**
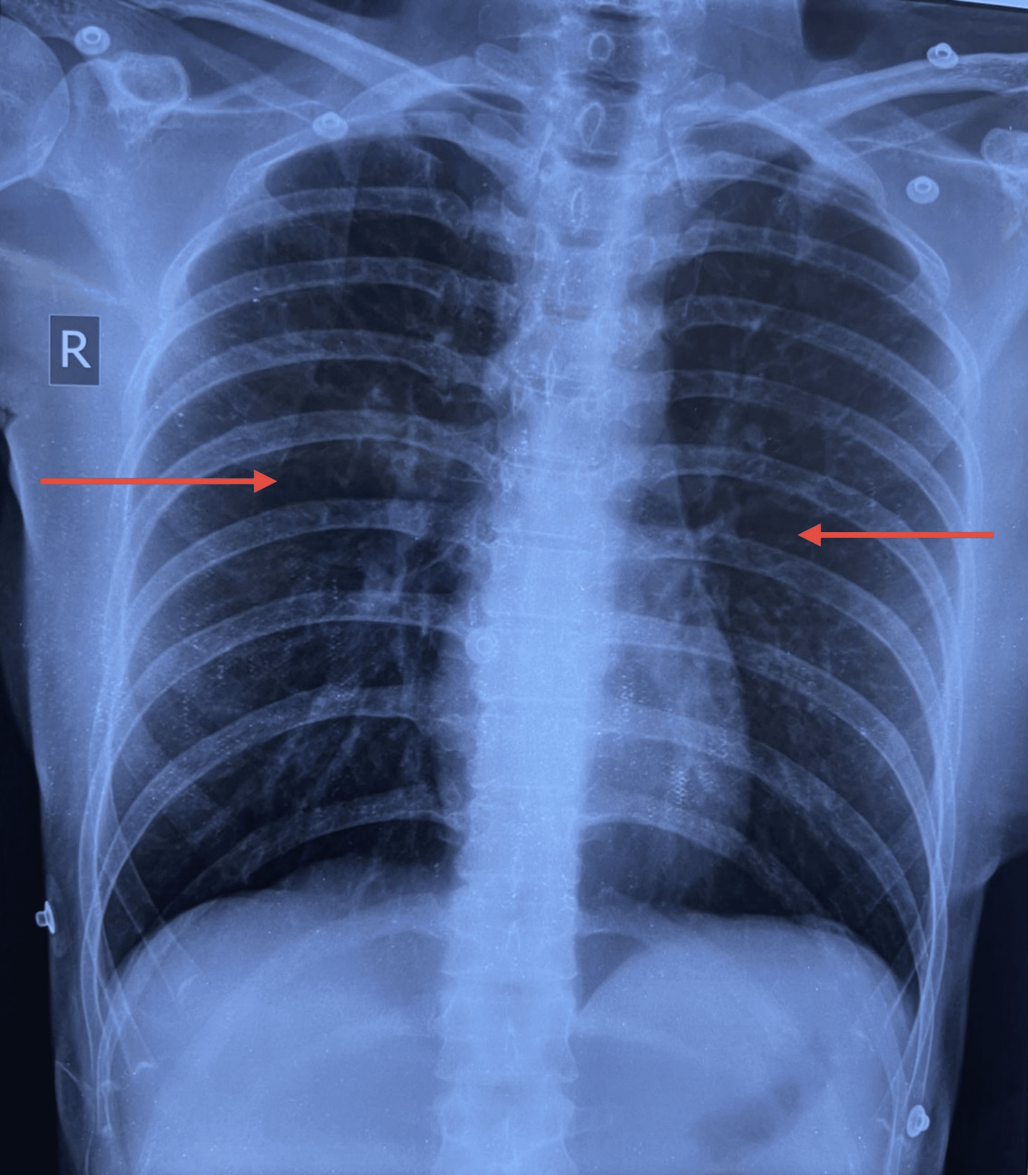
Chest X-ray on presentation showing clear lung fields without infiltrates(red arrows), normal cardiac silhouette, and no evidence of pulmonary edema or effusion.

A chest X-ray (Figure 3) was obtained, which revealed clear lung fields with no evidence of infiltrates, consolidation, pulmonary edema, or cardiomegaly.

Baseline laboratory investigations revealed that hematological parameters were within normal limits. Serum sodium was slightly reduced at 132 mmol/L, indicating mild hyponatremia. Serum creatinine was also mildly below the reference range at 0.58 mg/dL, which could be attributed to reduced baseline muscle mass or early hemodilution following fluid resuscitation. Potassium and chloride levels were within the normal range. These findings are summarized in Table 3.

**Table 3:** Hematological and Biochemical Parameters on Admission.

Parameter	Value	Reference Range
WBC Count	8.1 thou/µL	4.0 – 10.0 thou/µL
RBC Count	5.10 mil/µL (High)	3.8 – 4.8 mil/µL
Hemoglobin	12.8 g/dL	12.0 – 15.0 g/dL
Hematocrit	41.2 %	36 – 46 %
MCV	81 fL	80 – 100 fL
MCH	25.2 pg (Low)	27.0 – 32.0 pg
MCHC	31.1 g/dL (Low)	31.5 – 34.5 g/dL
Mentzer Index	15.9	–
RDW	13.8 %	11.6 – 14.0 %
Neutrophils	84 % (High)	40 – 80 %
Lymphocytes	11 % (Low)	20 – 40 %
Monocytes	3 %	2 – 10 %
Eosinophils	2 %	1 – 6 %
Basophils	0 %	<1 – 2 %
Platelet Count	217 thou/µL	150 – 410 thou/µL
Creatinine	0.58 mg/dL (Low)	0.60 – 1.10 mg/dL
Sodium	132 mmol/L (Low)	135 – 145 mmol/L
Potassium	4.1 mmol/L	3.5 – 5.0 mmol/L
Chloride	105 mmol/L	95 – 105 mmol/L

Urine analysis revealed a slightly hazy appearance with trace protein, 3-5 red blood cells, and 3-5 pus cells per high-power field, along with 5-7 epithelial cells. No casts, crystals, or bacteria were identified. Overall, the findings were not indicative of a urinary tract infection. A detailed summary is provided in Table 4.

**Table 4:** Urine Analysis on Admission.

Parameter	Value	Reference Range/Remarks
Color	Yellowish	–
Appearance	Slightly Hazy	–
pH	6.5	4.5 – 7.5
Specific Gravity	1.02	1.005 – 1.030
Glucose	Not Detected	Not Detected
Protein	Trace	Not Detected
Bilirubin	Not Detected	Not Detected
Urobilinogen	Normal	Normal
Nitrite	Not Detected	Not Detected
Pus Cells (WBC)	3–5 /HPF	0–5 /HPF
Epithelial Cells	5–7 /HPF	0–5 /HPF
Red Blood Cells	3–5 /HPF	Not Detected
Casts	Not Detected	Not Detected
Crystals	Not Detected	Not Detected
Bacteria	Not Detected	Not Detected
Amorphous Deposits	Not Detected	Not Detected

Coagulation parameters, including prothrombin time and international normalized ratio (INR), were within normal limits. Thyroid function was normal, with a TSH level of 1.230 µIU/mL. Allergy markers were assessed in view of the suspected anaphylactic episode. The total IgE level was markedly elevated at 413 kU/L, which supported the diagnosis of an IgE-mediated hypersensitivity reaction. The absolute eosinophil count remained within normal range, and dengue serology was negative. These results are outlined in Table 5.

**Table 5:** Summary of Coagulation Profile, Thyroid Function, Allergy, and Dengue Serology on Presentation.

Parameter	Value	Reference Range
Prothrombin Time (PT)	13.8 seconds	12.7 – 14.6 seconds
INR	1	0.00 – 1.40
Mean PT of Control Plasma	13.7 seconds	< 13.7 seconds
TSH (Ultrasensitive)	1.230 µIU/mL	0.27 – 4.20 µIU/mL
Total IgE	413.0 kU/L (High)	< 100 kU/L
Absolute Eosinophil Count	0.13 thou/µL	0.02 – 0.50 thou/µL
Dengue NS1 Antigen	Negative	–
Dengue IgG Antibodies	Negative	–
Dengue IgM Antibodies	Negative	–

Serum tryptase levels were not measured, as the patient presented more than two hours after symptom onset, which is beyond the optimal diagnostic window for reliable interpretation. Ultrasound of the abdomen and pelvis revealed no significant abnormalities.

In the ICU, supportive management was continued, including intravenous corticosteroids, intravenous fluids, nebulized bronchodilators, proton pump inhibitors (PPIs), and careful monitoring of hemodynamic parameters and oxygenation status.

Over the next 24 hours, the patient showed progressive improvement, with resolution of respiratory distress, normalization of oxygen saturation on room air, and stabilization of hemodynamic parameters. Once stable, she was transferred to the ward for continued observation. She was later discharged in stable condition with a prescription for oral antihistamines once daily and oral corticosteroids twice daily for one week, along with clear instructions on allergen avoidance, recognition of anaphylaxis symptoms, and with a follow-up appointment scheduled one week later.

## Discussion

Anaphylaxis is a severe, sometimes fatal systemic hypersensitivity reaction that affects multiple organs, including the skin, respiratory, gastrointestinal, and cardiovascular systems. Effective therapy remains largely dependent on early detection and intramuscular (IM) epinephrine injection. However, in rare manifestations, such as substantial tachycardia without hypotension, postural dizziness, or even paradoxical hypertension, present clinical problems that call for meticulous decision-making to avoid delays in diagnosis and treatment [10].

In our case, the patient developed anaphylaxis after consuming an oral third-generation cephalosporin antibiotic. Though less often reported than penicillins, cephalosporins are known triggers of drug-induced anaphylaxis, typically in the context of intravenous administration due to rapid systemic exposure. While cephalosporins are recognized causes of drug-induced anaphylaxis, most cases are reported with intravenous administration due to rapid systemic exposure. Anaphylactic reactions following oral intake are exceptionally uncommon, which underscores the unusual and clinically significant nature of this case. Along with severe tachycardia in the absence of hypotension, the patient showed classic cutaneous and respiratory symptoms of anaphylaxis. This hemodynamic profile presented a therapeutic conundrum since the full intramuscular dose of epinephrine ran the danger of aggravating myocardial ischemia or tachyarrhythmia. Studies in the literature have highlighted the need for tailored risk assessment in such situations by recording cases of cephalosporin-induced anaphylaxis complicated by reverse Takotsubo cardiomyopathy and stress-induced myocardial dysfunction following adrenaline use in such scenarios [11,12].

Total and specific IgE, serum tryptase, and, if available, basophil activation testing (BAT) are all part of the routine laboratory workup for suspected anaphylaxis. These investigations are particularly useful in atypical or delayed presentations, as they help confirm mast cell activation and identify potential triggers. In our case, total IgE was markedly elevated, supporting an atopic predisposition. However, specific IgE testing, serum tryptase, and BAT could not be performed due to limited availability and delayed presentation beyond the ideal diagnostic window. Despite this, the diagnosis of anaphylaxis remained clinically evident, based on the acute onset of symptoms, temporal association with the suspected drug, and response to treatment. However, the absence of these tests does not rule out anaphylaxis when the clinical features and timing clearly point to the diagnosis [13]

The first-line treatment for anaphylaxis is epinephrine, which is usually injected intramuscularly into the mid-anterolateral thigh at a dose of 0.01 mg/kg, up to a maximum of 0.5 mg in adults [14]. A total intramuscular dose of up to 1.5 mg is typically regarded as safe before switching to intravenous infusion or escalation of therapy, and the dose may be repeated every 5 to 15 minutes if there is no appropriate clinical response. The ideal route of administration is intramuscular because of its quick absorption and lower risk of cardiovascular problems. Despite being generally well tolerated, epinephrine can occasionally result in arrhythmias or myocardial infarction, palpitations, and tachycardia, particularly when administered intravenously or to people who already have cardiovascular illnesses [15].

In this instance, the patient exhibited classic features of anaphylaxis, accompanied by marked tachycardia and normotension, creating a clinical dilemma regarding the standard intramuscular administration of epinephrine. Concerns were raised about the potential for cardiovascular instability, including arrhythmias, with conventional dosing. Given the severity of symptoms and under continuous cardiac monitoring, a microdosed intravenous epinephrine strategy was cautiously employed. While this approach is not currently endorsed by major anaphylaxis guidelines and remains an off-label intervention, it reflects a risk-balanced, individualized decision tailored to the patient’s hemodynamic profile. Previous reports have documented serious complications such as atrial fibrillation following inadvertent or undiluted intravenous epinephrine use, underscoring the need for extreme care when considering this route [16].

In this case, the patient’s hemodynamic profile and the clinical judgment prompted us to use a microdosed intravenous epinephrine. This proved to be successful in controlling the symptoms without compromising cardiovascular function, therefore stressing the need for customized treatment plans for anaphylaxis management.

Adjunctive therapies in anaphylaxis include antihistamines, corticosteroids, and bronchodilators. Antihistamines may help reduce cutaneous symptoms but have no role in treating airway, breathing, or circulatory compromise. Corticosteroids have a delayed onset and are not effective for immediate symptom control, but may be considered to reduce the risk of biphasic reactions. Bronchodilators can be useful in patients with lower airway symptoms, such as wheeze, but do not address upper airway obstruction or circulatory collapse. These adjuncts should never delay or replace intramuscular epinephrine, which remains the cornerstone of anaphylaxis management [17].

Emerging alternatives to intramuscular epinephrine are gaining attention in anaphylaxis management. Notably, needle-free delivery systems such as nasal sprays have been developed to provide rapid, non-invasive administration. These innovations may improve treatment accessibility, particularly for patients with needle phobia or in settings where injections are not feasible. The latest FDA approval of a nasal epinephrine spray for emergency use further highlights the evolving landscape of anaphylaxis care and its emphasis on timely, user-friendly interventions [18].

Anaphylaxis can lead to major complications, including airway obstruction, cardiovascular collapse, and death if not promptly recognized and treated. Cardiovascular compromise may arise from systemic vasodilation, plasma leakage, and coronary artery spasm. In some cases, mast cell mediators trigger myocardial ischemia through mechanisms described in Kounis syndrome, even in patients without underlying cardiac disease [19].

Prevention of future episodes is a critical component of anaphylaxis management. Identifying and avoiding known triggers, documenting drug allergies clearly in medical records, and referring patients for allergist evaluation for further testing and risk stratification can reduce recurrence. Educating patients on early symptom recognition and equipping them with an epinephrine auto-injector, such as an EpiPen, for immediate self-administration during future episodes forms an essential safety net. Correct training on its use and emphasizing timely administration is crucial, as early epinephrine remains the most important intervention to reduce morbidity and mortality in anaphylaxis [20].

This case emphasizes the need for clinical discretion in controlling anaphylaxis in cases when the hemodynamic profile deviates from usual trends. In scenarios where patients present with severe tachycardia but maintain normal blood pressure, strict adherence to standard intramuscular epinephrine dosing may carry unnecessary risk. A carefully titrated, microdiluted intravenous approach proved effective in this instance, offering symptom relief without any cardiovascular compromise. When protocol-led care may introduce harm, an individualized strategy guided by patient physiology becomes essential. This case adds a great contribution to the developing knowledge of complex anaphylaxis management since there is little literature addressing such presentations.

## Conclusions

This case underscores the importance of clinical judgment in managing anaphylaxis when patients present with atypical hemodynamic profiles. In scenarios where significant tachycardia is observed without accompanying hypotension, the standard intramuscular dose of epinephrine may carry additional cardiovascular risks. In our patient, a carefully titrated microdosed intravenous epinephrine approach, administered under continuous monitoring, led to rapid clinical improvement without complications. This tailored strategy highlights the potential role of individualized treatment in select cases where guideline-directed therapy may not be ideal. This report adds to the limited literature addressing anaphylaxis management in normotensive patients with marked tachycardia and reinforces the value of prevention through allergen avoidance and patient education on early symptom recognition. However, it is important to acknowledge that microdosed intravenous epinephrine remains off-label in this context and is not currently recommended in standard guidelines. As such, further studies and real-world data are needed to better define its safety and efficacy in similar clinical scenarios.
